# Prefrontal Responses to Odors in Individuals With Autism Spectrum Disorders: Functional NIRS Measurement Combined With a Fragrance Pulse Ejection System

**DOI:** 10.3389/fnhum.2020.523456

**Published:** 2020-10-08

**Authors:** Mingdi Xu, Yasuyo Minagawa, Hirokazu Kumazaki, Ken-ichi Okada, Nozomi Naoi

**Affiliations:** ^1^Faculty of Letters, Keio University, Tokyo, Japan; ^2^Center of Life-Span Development of Communication Skills, Keio University, Yokohama, Japan; ^3^Global Centre for Advanced Research on Logic and Sensibility, Keio University, Tokyo, Japan; ^4^National Center of Neurology and Psychiatry, Tokyo, Japan; ^5^Faculty of Science and Technology, Keio University, Yokohama, Japan; ^6^Division of Arts and Sciences, College of Liberal Arts, International Christian University, Tokyo, Japan

**Keywords:** autism spectrum disorders (ASD), olfactory function, working memory, attention, detection threshold, dorsolateral prefrontal cortex (DLPFC), functional near-infrared spectroscopy (fNIRS)

## Abstract

Individuals with autism spectrum disorders (ASD) are impaired not only in social competencies but also in sensory perception, particularly olfaction. The olfactory ability of individuals with ASD has been examined in several psychophysical studies, but the results have been highly variable, which might be primarily due to methodological difficulties in the control of odor stimuli (e.g., the problem of lingering scents). In addition, the neural correlates of olfactory specificities in individuals with ASD remain largely unknown. To date, only one study has investigated this issue using functional magnetic resonance imaging (fMRI). The present study utilized a sophisticated method−a pulse ejection system−to present well-controlled odor stimuli to participants with ASD using an ASD-friendly application. With this advantageous system, we examined their odor detection, identification, and evaluation abilities and measured their brain activity evoked by odors using functional near-infrared spectroscopy (fNIRS). As the odor detection threshold (DT) of participants with ASD was highly variable, these participants were divided into two groups according to their DT: an ASD-Low DT group and an ASD-High DT group. Behavioral results showed that the ASD-High DT group had a significantly higher DT than the typically developing (control) group and the ASD-Low DT group, indicating their insensitivity to the tested odors. In addition, while there was no significant difference in the odor identification ability between groups, there was some discrepancy between the groups’ evaluations of odor pleasantness. The brain data identified, for the first time, that neural activity in the right dorsolateral prefrontal cortex (DLPFC) was significantly weaker in the ASD-High DT group than in the control group. Moreover, the strength of activity in the right DLPFC was negatively correlated with the DT. These findings suggest that participants with ASD have impairments in the higher-order function of olfactory processing, such as olfactory working memory and/or attention.

## Introduction

Individuals with autism spectrum disorders (ASD) generally have unusual sensory awareness, showing either hyper- or hypo-responsiveness to various sensory modalities, including olfaction (Kientz and Dunn, 1997; Rogers et al., 2003; Rogers and Ozonoff, 2005; Schreck and Williams, 2006; Ben-Sasson et al., 2009; Wiggins et al., 2009). Accumulating evidence indicates that individuals with ASD experience more sensory disturbances than typically developing (TD) individuals or individuals with intellectual disabilities (Leekam et al., 2007; Tomchek and Dunn, 2007). Particularly, an aberrant response to smell has been repeatedly reported in the ASD population (Schecklmann et al., 2013; Martin and Daniel, 2014; Rozenkrantz et al., 2015; Endevelt-Shapira et al., 2018) and has been suggested as a prominent criterion for distinguishing individuals with ASD from those with other developmental disorders (Rogers et al., 2003; Leekam et al., 2007; Schoen et al., 2009).

### Sensory Psychophysical Studies of Olfactory Perception in ASD

Previous studies have found that over 50% of sampled children with ASD had unusual smell and/or taste sensitivity (Schoen et al., 2009; Lane et al., 2010). Moreover, olfactory problems have been suggested to be a good predictor of social deficiency in individuals with ASD (Liss et al., 2006; Bennetto et al., 2007; Hilton et al., 2007; Lane et al., 2010), and olfactory alterations have been suggested as an early marker for ASD (Brewer et al., 2006; Hrdlicka et al., 2011).

Odor is a very powerful sensory modality, capable of eliciting strong emotional reactions (Soudry et al., 2011) and episodic memory (Saive et al., 2014) in humans and can serve as a potent cue for both social and cognitive development in children with ASD (Parma et al., 2013; Woo et al., 2015). Despite its importance, relatively few studies have investigated the sense of smell in individuals with ASD. Moreover, these studies have reported conflicting results across a variety of domains. Specifically, several studies found that individuals with ASD are impaired in the ability to identify odors (Suzuki et al., 2003; Bennetto et al., 2007; May et al., 2011; Galle et al., 2013; Wicker et al., 2016) compared with TD individuals, while other studies did not find a significant difference between the two groups (Brewer et al., 2008; Dudova et al., 2011; Addo et al., 2017). Similarly, some studies suggested preserved odor sensitivity in individuals with ASD (Suzuki et al., 2003; Tavassoli and Baron-Cohen, 2012; Galle et al., 2013; Addo et al., 2017), whereas other studies reported either enhanced (Ashwin et al., 2014) or decreased odor sensitivity (Dudova et al., 2011; Kumazaki et al., 2016). In addition, Hrdlicka et al. (2011) found atypical hedonic responses to odor stimuli in individuals with ASD; however, Galle et al. (2013) and Addo et al. (2017) did not find such responses. Likewise, while Kumazaki et al. (2019) demonstrated decreased odor adaptation in children with ASD as compared to TD children, Tavassoli and Baron-Cohen (2012) reported similar odor adaptation in adults with ASD and TD adults. This is in contrast to the well-established literature on abnormalities in vision (Simmons et al., 2009), audition (O’Connor, 2012), and touch (Puts et al., 2014) in individuals with ASD.

The discrepancies between studies might be due to many factors including large variability in participants’ age (from childhood to adulthood) and ASD subtype [e.g., high functioning autism (HFA), Asperger’s syndrome (AS), and pervasive developmental disorder (PDD)], as well as in tests [e.g., Sniffin’ Sticks (Hummel et al., 1997), University of Pennsylvania Smell Identification Test (UPSIT; Doty et al., 1984b), alcohol sniff test (AST; Davidson and Murphy, 1997), and custom-made tests] and/or odors used (e.g., n-butanol, alcohol, custom-made), making direct comparison difficult.

### Neuroimaging Studies of Olfaction in ASD

Studies of sensory perception in ASD have predominantly investigated auditory and visual differences in individuals with ASD while olfactory differences are least studied (Martin and Daniel, 2014; Kumazaki et al., 2019), particularly at the neural level.

Non-invasive neuroimaging modalities such as magnetic resonance imaging (MRI) have expanded the knowledge of olfactory dysfunction in humans (Han et al., 2019). Anatomically, dysfunction in areas often implicated in ASD, such as the orbitofrontal and medial temporal areas including the amygdala, may be responsible for olfactory deficits (Amaral et al., 2008; Stanfield et al., 2008; Tonacci et al., 2017). In addition, a bioinformatics study exploring the genetic heterogeneity of olfaction in individuals with ASD suggested that four brain regions are critically related to ASD pathogenesis: the olfactory bulb, occipital lobe, prefrontal cortex, and pituitary (Kumar et al., 2011).

Functional neuroimaging studies investigating the neural basis of olfactory processing in individuals with ASD are scarce. To the best of our knowledge, only two recent functional MRI (fMRI) studies have probed the neural responses of individuals with ASD to odors (Koehler et al., 2018; Stickel et al., 2019). Koehler et al. (2018) examined the neural response of participants with ASD (18 participants aged 29.5 ± 2.51 years with HFA and AS; two women) to odor detection and identification in the olfactory cortex including the piriform cortex, amygdala, and orbitofrontal cortex (OFC). They found impaired odor detection and odor identification in participants with ASD and reported, for the first time, significantly attenuated odor-induced brain response in the piriform cortex as well as a trend toward decreased activity in the OFC in participants with ASD compared to TD controls. Stickel et al. (2019) did not directly examine brain function in individuals with ASD in response to odors; instead, they investigated olfactory- and auditory-visual integration (essentially multisensory integration). Similar neural networks, including the medial and inferior frontal cortices, were found to be involved in the multisensory integration processes, which were not significantly different between participants with ASD and their matched TD counterparts.

With functional near-infrared spectroscopy (fNIRS), much knowledge has been accumulated about sensory perception, including olfactory processing (Ishimaru et al., 2004a,b; Harada et al., 2006; Kobayashi et al., 2009; Takakura et al., 2011). For example, Ishimaru et al. (2004a, b), Harada et al. (2006), and Kobayashi et al. (2009) reported activation (increased oxy-Hb concentration) of the most anterior part of the prefrontal areas in response to olfactory stimuli and suggested that such hemodynamic responses might reflect activity in the OFC corresponding to the secondary olfactory cortex (Zatorre et al., 1992; Saive et al., 2014). Similar prefrontal activity, primarily in the frontal pole (FP) and dorsolateral prefrontal cortex (DLPFC), was reported by Takakura et al. (2011) and was suggested to indicate attention and working memory related to the odor detection task. Nevertheless, an important knowledge gap remains, in that none of these fNIRS studies included individuals with ASD in spite of its amenability to the ASD population.

Considering abnormality in executive functions including attention and working memory in individuals with ASD (Russell, 1997; Hill, 2004; Kenworthy et al., 2008; Travers et al., 2011), it is possible that their brain activity in the region responsible for these functions (i.e., the DLPFC; Duncan and Owen, 2000; Levy and Goldman-Rakic, 2000) is different from that of TD individuals in tasks requiring executive functions. This possibility might also partially account for the olfactory hyposensitivity in younger individuals with ASD, which was found in the meta-analysis of Larsson et al. (2017). This analysis has suggested that younger individuals with ASD (<30 years) tend to show olfactory hyposensitivity, whereas older individuals with ASD (>35 years) do not. Since the development of prefrontal region continues until early adulthood (Diamond, 2002), the immaturity of this region in younger individuals with ASD may result in their olfactory hyposensitivity.

### Heterogeneity in Odor Sensitivity in Individuals With ASD and Methodological Obstacles to Olfactory Stimulation

Autism spectrum disorders is characterized by a high degree of heterogeneity across individuals (Jeste and Geschwind, 2014). In addition to the large variability in individuals’ intelligence and their sensory and attentional capacities, neuroimaging studies have demonstrated heterogeneity in both structural and functional brain abnormalities among individuals with ASD (Anagnostou and Taylor, 2011; Dichter, 2012). It is possible that, apart from methodological discrepancies, divergent findings among studies investigating olfactory perception in individuals with ASD might be due to a complex blend of these factors. When assessing odor-evoked neural responsiveness in individuals with ASD, it is fundamental to assess whether and to what degree the used odors can be actually perceived. Thus, to evaluate functionality of the olfactory system, it is critical to examine the odor detection threshold (DT) of each participant. In fact, Koehler et al. (2018) tested odor detection and identification abilities in ASD participants before scanning sessions but did not directly correlate these datasets.

As mentioned above, the investigation of olfactory perception in individuals with ASD has yielded inconsistent results, and methodological obstacles might be one of the most significant reasons for such results. Most of these studies have used UPSIT, AST, and Sniffin’ Sticks, for which control of odor granularity is challenging due to the problem of lingering scents (Fukasawa et al., 2013). As olfaction is a highly adaptable sensory modality, measurements of olfactory ability can be compromised when the odor stimuli remain in the air (Kumazaki et al., 2016). In addition, prolonged odor exposure will lead to odor adaptation, which decreases the measurement accuracy (Dalton and Wysocki, 1996). To solve these problems, we developed the Fragrance Jet for Medical Checkup (Keio University) that uses a pulse ejection system (Fukasawa et al., 2013) and which has been reliably standardized and successfully used in our previous work (Kumazaki et al., 2016, 2018a,b, 2019). It employs an identical technique as a basic inkjet printer, using a very small quantity of an odorant to emit tiny droplets of scent. This technique can be fine-tuned with respect to the amount and time of exposure to reduce lingering scents, allowing precise assessment of the olfactory function. More specifically, by modulating the number of simultaneous ejections (NSE), the ejection quantity per unit time (EQUT) can be adjusted, which, together with the ejection time (ET), determines the intensity of ejected odor (please see Fukasawa et al., 2013 for details). Instead of preparing various concentrations of scent beforehand as in conventional olfactory measurement techniques, our approach makes measurement by only changing the NSE. Using a very small quantity of an odor reduces lingering scents and avoids odor adaptation (Sato et al., 2008), which is an important confounding factor during the assessment of olfaction. However, because many parts of the instrument are metal, this type of instrument cannot be used with fMRI scanning but can be combined with fNIRS.

To address the aforementioned issues, the present study uses fNIRS to investigate cerebral activation in olfactory processing in young adults with ASD and TD young adults. The primary aim of this study was to examine the feasibility of combined usage of a fragrance pulse ejection system for presenting odor stimuli with fNIRS system. The secondary aim was to reveal differential functions of the prefrontal region in odor processing in participants with ASD and TD participants by carefully examining the relationship between odor sensitivity and associated neuronal responses in participants with ASD. We predict that both odor perception and odor-induced neural function are impaired in participants with ASD and that their brain activity is correlated with their odor sensitivity.

## Materials and Methods

### Participants

The present study was approved by the ethics committee of the Keio University, Faculty of Letters (No. 16028). The participants were young adults with ASD and TD young adults. Twenty-five participants with ASD (19 males, 18–24 years old, mean age: 20.50 years) and 16 TD participants (13 males, 19–24 years old, mean age: 21.33 years) volunteered for the study. The exclusion criteria for both participants with ASD and TD participants included organic smell disturbance, nasal problems, diagnosed psychiatric conditions, and a history of head injury. After a complete explanation of the study, all volunteers and their parents agreed to participate in the study and provided written informed consent.

The participants with ASD were diagnosed by psychiatrists using the Diagnostic and Statistical Manual of Mental Disorders (DSM-5; American Psychiatric Association, 2013) criteria and the standardized criteria taken from the Diagnostic Interview for Social and Communication Disorders (DISCO; Leekam et al., 2002) at the time of enrollment in the study. The TD participants had no history or evidence of ASD, but they were tested for autism traits using autism spectrum quotient (AQ; Baron-Cohen et al., 2001). The psychiatrists also categorized participants with ASD into three subtypes based on DISCO: AS, autistic disorder (AD), and pervasive developmental disorder not otherwise specified (PDD-NOS; see Table 1).

**TABLE 1:** Descriptive characteristics of the ASD and control (TD) groups.

**Characteristics**	**ASD-Low DT (*n* = 12) (ME, SD)**	**ASD-High DT (*n* = 13) (ME, SD)**	**Control (*n* = 13) (ME, SD)**	**Statistics**
Age in years	19.8 (1.7)	19.9 (2.0)	20.6 (1.7)	*F*(2,35) = 0.889, *p* = 0.42
Gender (M:F)	9:3	10:3	10:3	χ^2^ (2) = 0.017, *p* = 0.99
Type of ASD	1 AD, 7 AS, 4 PDD-NOS	3AD, 1 AS, 9 PDD-NOS		
Full scale IQ	77.8 (10.8)	65.2 (12.7)	115.9 (7.6)	*F*(2,35) = 79.83, *p* < 0.001 Control vs. ASD-Low DT: *p* < 0.001 Control vs. ASD-High DT: *p* < 0.001 ASD-Low DT vs. ASD-High DT: *p* = 0.018
AQ-J			17.5 (9.6)	
CARS-TV	32.7 (2.0)	31.8 (2.7)		*t* (23) = 0.945, *p* = 0.36
DT				See section “Behavior Results”
Rose	49.2 (7.0)	108.5 (6.7)	39.2 (6.7)	
Mint	27.5 (16.0)	82.3 (49.9)	28.5 (17.7)	
Mean	38.3 (9.4)	95.4 (20.4)	33.9 (15.8)	
				Two-way ANOVA: Main effect of odor: *F*(1,35) = 6.51, *p* = 0.02 Rose > Mint Main effect of group: *F*(2,35) = 59.24, *p* < 0.001 ASD-High DT > Control (*p* < 0.001) ASD High-DT > ASD Low-DT (*p* < 0.001) No interaction: *F*(2,35) = 0.37, *p* = 0.70

*ASD, autism spectrum disorders; TD, typical development; DT, odor detection threshold; ME, mean; SD, standard deviation; Parentheses indicate SD. M, male; F, female; AD, autistic disorder; AS, Asperger’s syndrome; PDD-NOS, pervasive developmental disorder not otherwise specified; IQ, intelligence quotient. We did not obtain IQ from one participant with ASD due to his low motivation; AQ-J, autism spectrum quotient, Japanese version. Higher scores indicate a greater number of ASD-specific behaviors; CARS-TV, Childhood Autism Rating Scale-Tokyo Version. Higher scores indicate high autistic symptoms.*

To assess autistic traits of participants with ASD, the Childhood Autism Rating Scale-Tokyo Version (CARS-TV) was used. The CARS-TV is the Japanese version of the CARS (Schopler et al., 1980)—one of the most widely used scales to evaluate the degree and profiles of autism in children—which has been shown to have satisfactory reliability and validity (Kurita et al., 1989; Tachimori et al., 2003). We did not use AQ for participants with ASD because it chiefly examines autistic tendency in neurotypical individuals. In addition, as CARS data for the ASD groups had been provided to us, we refrained from applying further questionnaires to them to avoid taking them too much time.

Intelligence testing in both participants with ASD and TD participants was performed using the Wechsler Intelligence Scale for Children—Fourth Edition (WISC-IV; Wechsler, 2003) or Wechsler Adult Intelligence Scale—Third Edition (WAIS-III; Dumont and Willis, 2008). We did not obtain IQ from one participant with ASD due to his low motivation. In addition, both participants with ASD and TD participants were tested for their perceptual traits using a sensory profile (Dunn et al., 1999). The participants’ demographic information is shown in Table 1.

### Procedure

**FIGURE 1:**
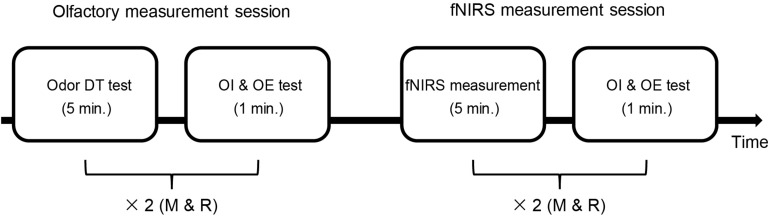
Time sequence of the whole experiment. There were two sessions: the olfactory measurement session and the fNIRS measurement session. The olfactory measurement session included an odor detection threshold (DT) test and an odor identification (OI) and odor evaluation (OE) test. First, the odor DT test was performed for each odor (rose and mint, as shown with “×2”). Then, both odors were used once in the OI and OE test. In the fNIRS measurement session, the OI and OE test was performed after the fNIRS measurement using each odor (two sets of fNIRS measurement). The time used is shown in minute (min.) M, mint; R, rose.

Both the participants with ASD and the TD participants completed an olfactory measurement session that included assessment of odor DT, odor identification, and odor evaluation. The participants subsequently received an fNIRS assessment, after which they underwent odor identification and evaluation again. The details of the procedure can be seen in Figure 1.

### Olfactory Measurement

#### Odor Presentation

**FIGURE 2:**
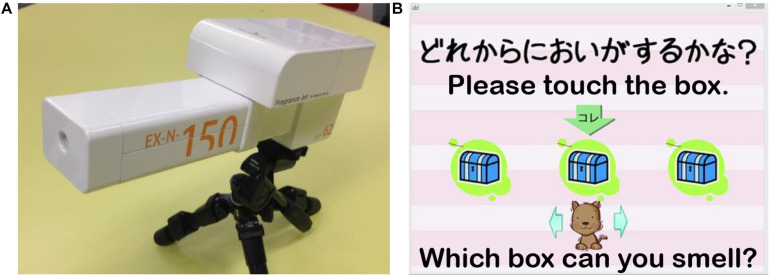
**(A)** The olfactory display using a pulse ejection system. **(B)** A screenshot of the application for odor detection threshold (DT) measurement designed for participants with ASD (Matsuura et al., 2014).

**FIGURE 3:**
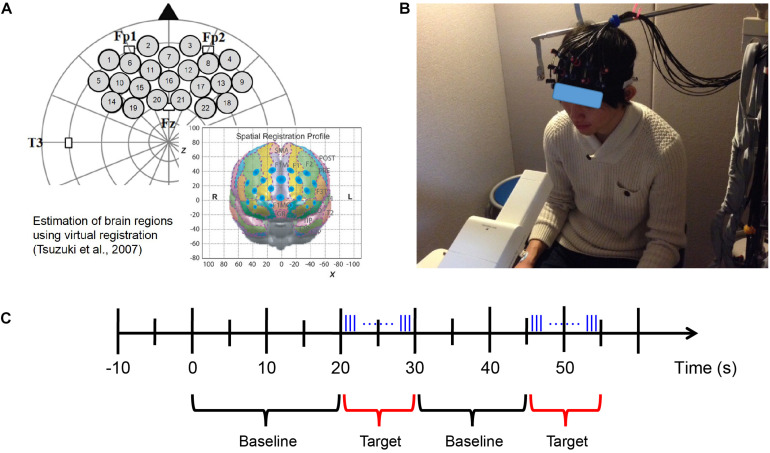
fNIRS measurement. **(A)** Probe set and position on the brain (Xu et al., 2017). **(B)** fNIRS experimental environment (informed consent was obtained from the participant shown in the figure). **(C)** Block design of fNIRS measurement. Odor stimuli were emitted during the 10 s of the target period. The blue vertical lines represent the pulses of odor stimuli presented by the fragrance pulse ejection system. The odor stimuli were emitted for 200 ms (200/0.667 = 300 pulses) at a time, and the inter-stimulus interval was 80 ms.

Two types of odors were used for each olfactory measurement: a simple chemical β-phenylethyl alcohol that smells like rose and a natural fragrance of mint. Odorants were diluted to 5% using water and little ethanol to adjust their adhesiveness. In our preliminary experiment, we tried many odors and found that the odors of rose and mint were most suitable for obtaining consistent brain responses from participants, which is important for the success of the fNIRS experiment. The experiment room was well-ventilated to prevent lingering scents. Olfactory measurements were performed using an olfactory display (Figure 2A), which uses a pulse ejection system (Fukasawa et al., 2013) and can measure and quantify odor DT with high precision. It uses an ejection head to produce scent droplets from tiny holes. The device has one large tank and three small tanks. We used the large tank for DT measurement and the small tanks for odor identification and odor evaluation measurements. There are 255 tiny holes in the ejection head connected to the large tank and 127 tiny holes in the head connected to the small tanks. These tiny holes can emit scents simultaneously. The average ejection quantity from a single hole was referred to as the unit average ejection quantity, which is 7.3 pL for the large tank and 4.7 pL for the small tanks. The intensity of ejected odor is determined by two parameters: EQUT and ET. The EQUT can be adjusted by modulating the NSE (0 ∼ 255 for the large tank, and 0 ∼ 127 for the small tanks). Ejections can be controlled in pulses of 667 μs; ET determines the number of ejected pulses (ET/667; please see Fukasawa et al., 2013 for details). Participants sat in front of the pulse ejection system from a distance of approximately 20 cm (Figure 3B).

#### Odor DT

The task was designed to be as simple as possible so that participants with ASD could concentrate and complete the measurement without making verbal responses. To this end, we used a game-like application developed in our previous work (Matsuura et al., 2014) to perform the experiment using a touch panel (Figure 2B). In each trial, three boxes were shown on the display of the touch panel, each of which contained an odor stimulus. The three stimuli were arranged pseudo-randomly; one stimulus was scented while the other two were odorless. When the participants clicked one of the three boxes, an odor was emitted 3.0 s later. The participants were allowed to click each box up to two times and were asked to identify the box that contained the odor stimulus (triple forced-choice). Before measurement, we confirmed that all participants understood the instructions. Based on the results of preliminary tests and our earlier studies (Fukasawa et al., 2013; Kumazaki et al., 2016), we began the measurement with an NSE of 60 and an ET of 200 ms. A specific measurement algorithm that employs a binary search (Fukasawa et al., 2013; Kumazaki et al., 2019) was used to determine the participants’ DT (Supplementary Figure 1). For the first trial, when a participant made an error (selecting a box that contained an odorless stimulus), the NSE increased by 50%; once two consecutive trials were successful, the NSE decreased by 50%. For the rest of the trials, the increment or decrement unit of the NSE was 10. The maximum and minimum values of NSE were 120 and 10, respectively. These values were also based on the results of preliminary tests and our earlier study on olfaction in individuals with ASD using the same odor presentation device (Kumazaki et al., 2019). All participants’ measurements were completed in approximately 5 min. The DT was generated after the procedure was completed. This odor DT test was conducted once for both rose and mint odors.

#### Odor Identification and Odor Evaluation

There were two sessions for measuring odor identification and odor evaluation: the olfactory measurement session and the fNIRS measurement session (see Figure 1). Both rose and mint odors were presented to the participants once in each session, and the sequence of the two odors in each session was randomized among participants. In the olfactory measurement session, an odor stimulus at an intensity of 120 NSE was presented to a participant for an ET of 200 ms. Subsequently, the participant was asked to verbally answer what kind of odor he/she smelt (odor identification) and how pleasant they thought the smell was (odor evaluation) if he/she had perceived it. For the odor identification test, we used a free identification paradigm in which no odor descriptor was provided to the participants. The participants got 3 points if they explicitly named the odor (i.e., rose or mint). They got 2 points if they answered flower or herb. They got 1 point if they generalized the odor as sweet or cooling. Otherwise, they got 0 points. For odor evaluation, the participants were asked to verbally rate the pleasantness of the odor from 1 (very pleasant) to 5 (very unpleasant) for each trial. The details of the odor identification and odor evaluation tests in the fNIRS measurement session are provided in the following subsection.

### fNIRS Measurement

Hemodynamic responses in the prefrontal region were recorded using a multichannel NIRS system (ETG-7000, Hitachi Medical Co., Japan). The system emits continuous near-infrared lasers with fixed wavelengths of approximately 780 and 830 nm. Lasers are modulated at different frequencies depending on the wavelengths and the channels and are detected using lock-in amplifiers (Watanabe et al., 1996). The device provides estimates of changes in hemoglobin (Hb) concentrations and oxygenation levels of the optical paths in the underlying brain regions between the nearest pairs of emitter and detector probes.

A silicon probe pad was used to arrange eight emitters and seven detector probes in a 3 × 5 rectangular lattice, forming 22 recording channels. Each pair of emitter and detector probes was separated by 30 mm. The 3 × 5 probe pad was placed on the participants’ prefrontal region (Figure 3A; Xu et al., 2017). Specifically, the bottom edge of the probes was placed in a direction horizontal to the line connecting T3, Fp1, Fp2, and T4 on the international 10–20 system, and the center of the channels was positioned across the nasion–inion line (Klem et al., 1999). This probe arrangement enabled the spatial estimation of localized cerebral activity based on the virtual registration method (Tsuzuki et al., 2007). After probe placement, the experimenter verified that each probe was in adequate contact with the scalp. Only after this verification were fNIRS recordings initiated.

During the fNIRS measurement, the participants were shown a silent video of running trains to help them keep their head position stable as much as possible while the odor was presented at a random time using a block design (Figure 3B). We aimed to use this approach (1) to maintain the distance from the odor emitting instrument to the participants’ nose and (2) to reduce motion artifact caused by head movement in the fNIRS data as has been used in neuroimaging experiments with passive task for young population (e.g., Dehaene-Lambertz, 2000; Minagawa-Kawai et al., 2009). These are important factors for evaluation of neural responses elicited by olfaction. Moreover, since the video was silent and monotonous, we presume that even if it would occupy the participants’ attention to some extent, the degree would not vary much among participants and stimulus conditions.

The length of the baseline period was randomized from 15 to 25 s; the target period was 10 s, within which the odor stimuli were emitted for 200 ms (200/0.667 = 300 pulses) at a time, and the inter-stimulus interval was 80 ms (Figure 3C). The fNIRS measurement also used the odors of mint and rose and executed eight blocks of odor stimulation for each odor. After completing the fNIRS measurement session using each odor, the participants were required to identify and evaluate the odor.

To minimize the influence of individual difference in breathing, we asked the participants to breathe at a fixed pace (inhale for 2 s and exhale for 3 s) in the preliminary experiment. We found that pace-controlled breathing led to so much physiological noise that it even concealed the neural responses induced by the odor stimulus itself. We therefore used various lengths of baseline period (15–25 s) to avoid the influence of regular breathing cycle and let the participants breathe at their normal pace during the target period of each block to minimize the impact of breathing as much as possible. In addition, the odor stimulus was presented to the participants continuously during the 10-s target period of each block and there were eight blocks in total. We chose a 10-s target period because natural breathing occurs once within 3–4 s in human adults (Dishman et al., 2000; Ragnarsdóttir and Kristinsdóttir, 2006) and this combined with a total of eight blocks enables all the participants to have inhaled the odor stimulus for several times during the fNIRS session.

### Behavioral Data Analysis

First, the participants with ASD were divided into two groups using a median split according to their DT scores. The participants with ASD with DT < 60 were labeled as the “ASD-Low DT” group and those with DT ≥ 60 were labeled as the “ASD-High DT” group. Next, their performance in odor identification and odor evaluation was also grouped. Two-way repeated measures analysis of variance (ANOVA) with the groups used as between-subject factors (control, ASD-Low DT, ASD-High DT) and the odors used as within-subject factors (mint, rose) was applied to the DT using IBM SPSS Statistics 25. For odor identification and evaluation, two-way repeated ordinal regression with cumulative link mixed models (CLMM; Christensen, 2015) was applied using R (R Core Team, 2018).

### NIRS Data Analysis

The NIRS data were preprocessed using Platform for Optical Topography Analysis Tools (POTATo) developed by Research and Development Group, Hitachi, Ltd., in a Matlab 7.7 environment (The MathWorks, Inc., Natick, MA, United States). Changes in the concentration of oxygenated (oxy-) Hb and deoxygenated (deoxy-) Hb were calculated from absorbance changes of 780 and 830 nm laser beams sampled at 10 Hz. For each participant, the raw oxy- and deoxy-Hb data in each channel were high-pass filtered at 0.0167 Hz to remove components originating from systematic fluctuations (Naoi et al., 2012). Blocks with motion artifacts were excluded [signal variations larger than two standard deviations (SD) from the mean over 0.2 s]. Any block containing oxy-Hb changes larger than 0.15 mM/mm within 0.2 s was discarded. The oxy-Hb and deoxy-Hb concentrations of the remaining baseline and target blocks were smoothened with a moving average of 5 s. To eliminate long-term signal trends due to systemic vascular factors, a first-degree baseline fit was estimated for each channel using the first 5 s and last 5 s of the analysis block.

For each group (control, ASD-Low DT, and ASD-High DT), the block analysis focused on a 25-s epoch composed of a 5-s prestimulus baseline period, a 10-s target period with odor stimulation, and a 10-s poststimulus period. The Hb concentrations of all artifact-free trials were averaged. Subsequently, a time course of the mean change in oxy-Hb and deoxy-Hb concentrations was compiled for each channel of each participant. These time courses for all the participants in each group were subsequently averaged to form time-dependent waveforms of the hemodynamic responses in each channel. Considering the slow characteristics of neural hemodynamic responses, a 10-s period starting 5 s after stimulus onset was regarded as the analysis window (10 ∼ 20 s of each block). A 5-s period immediately before stimulus onset was regarded as the time window for the baseline (0 ∼ 5 s of each block). A two-way repeated measures ANOVA with the group as the between-subject factor and odor as the within-subject factor was analyzed for the oxy-Hb concentration changes (the mean of oxy-Hb during the analysis window vs. the mean of oxy-Hb during the baseline) in each channel. A Bonferroni *post hoc* test was used to further evaluate any significant main effects. In addition, the means of oxy-Hb during the analysis window and the baseline period in each channel were analyzed by a paired *t*-test to identify the activated regions in response to the olfactory stimuli for each group. The brain regions underlying each channel were estimated using the virtual registration method for NIRS channels (Tsuzuki et al., 2007). For activated channels showing significant main effect of group, latency of the oxy-Hb peaks and mean amplitude of the deoxy-Hb and latency of the deoxy-Hb peaks within the analysis window were analyzed as well. Lastly, correlations between the oxy-Hb concentration changes and behavioral data (odor DT, odor identification, and odor evaluation), as well as the scores in the sensory profile, were assessed using Pearson correlation.

## Results

### Behavioral Results

There were 12 participants with ASD in the “ASD-Low DT” group and 13 participants in the “ASD-High DT” group. Three control participants were excluded from further data analysis because of technical problems with the odor presentation device.

**FIGURE 4:**
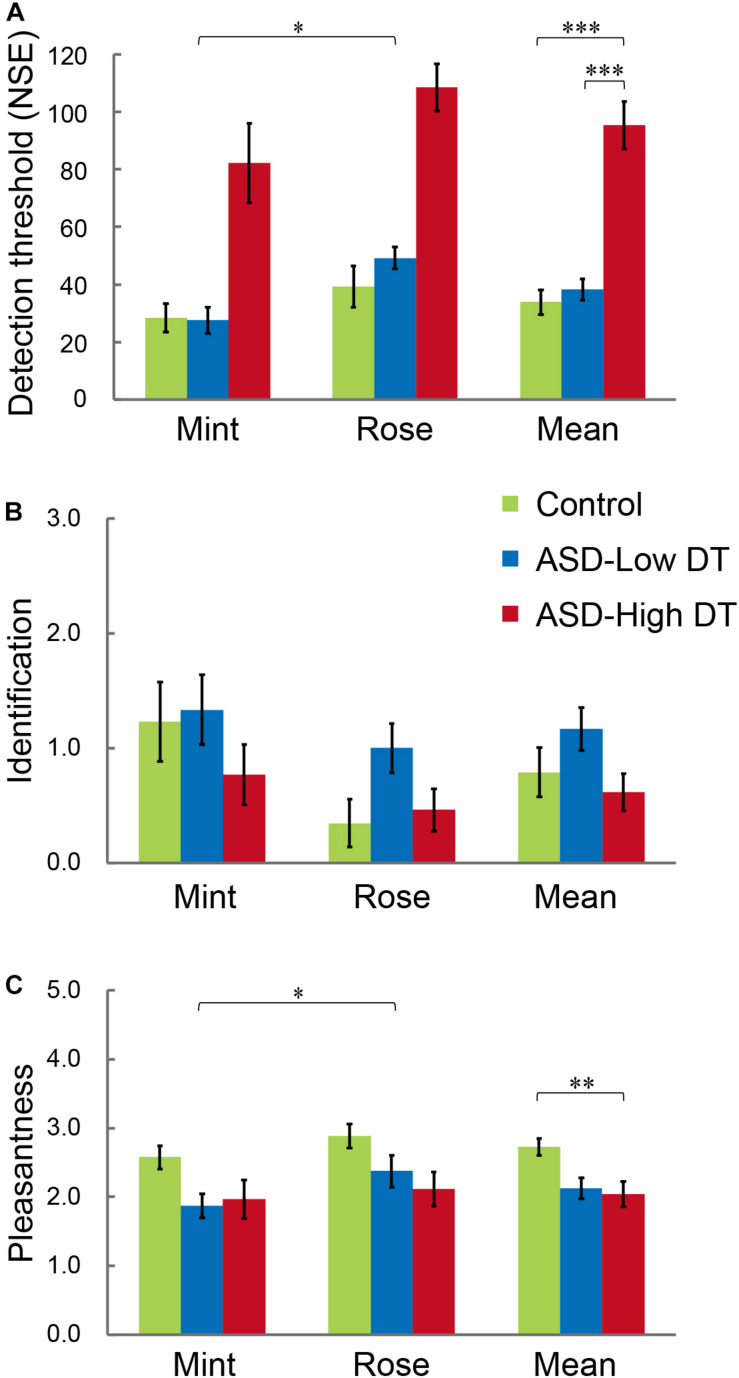
Performance of participants with ASD and TD control participants in the **(A)** odor detection threshold (DT), **(B)** odor identification, and **(C)** odor evaluation (pleasantness) tests. The ASD group was divided into “ASD-Low DT” (those DT < 60, 12 participants) and “ASD-High DT” (those DT ≥ 60, 13 participants) subgroups. Error bars represent 1 standard error. *p < 0.05; **p < 0.01; ***p < 0.001.

The results of DT (Figure 4A) showed higher DT for rose than for mint, and higher DT in the ASD-High DT group. This tendency was supported by two-way repeated measures ANOVA using odor (rose vs. mint) as the within-subject factor and group (control vs. ASD-Low DT vs. ASD-High DT) as the between-subject factor. Specifically, this analysis showed significant main effects of odor [*F*(1,35) = 6.51, *p* = 0.02] and group [*F*(2,35) = 59.24, *p* < 0.001]. There was no significant interaction effect between odor and group [*F*(2,35) = 0.37, *p* = 0.70]. For the significant main effect of odor, the DT (mean ± SD) for rose (66.05 ± 39.08) was significantly higher than for mint (46.58 ± 40.82). A Bonferroni *post hoc* analysis for the significant main effect of group revealed that the DT was significantly higher in the ASD-High DT group (95.38 ± 42.35) than in the control group (33.85 ± 22.29; *p* < 0.001) and the ASD-Low DT group (38.33 ± 18.10; *p* < 0.001), indicating significantly lower odor sensitivity in the ASD-High DT group (Figure 4A).

Two-way repeated ordinal regression with CLMM was applied to the performance of odor identification and odor evaluation. For odor evaluation, a higher rate indicates unpleasantness while a lower rate indicates pleasantness. After comparisons of a series of models with different fixed and random effects, the most appropriate model evaluated by the likelihood ratio test was accepted. For odor identification, there were no significant main effects or interactions (Figure 4B). For odor evaluation (rating of pleasantness; Figure 4C), the most appropriate model included the odor (rose vs. mint) and group (control vs. ASD-Low DT vs. ASD-High DT) as fixed effects, and the intercepts for odor and NIRS (with or without NIRS measurement) and by-subject random slopes as random effects. Both odor [χ^2^ (1) = 6.03, *p* = 0.014] and group [χ^2^ (2) = 7.02, *p* = 0.030] significantly affected odor evaluation. Specifically, the odor of mint (2.14 ± 0.81) was rated as significantly more pleasant than that of rose (2.46 ± 0.83). *Post hoc* analysis revealed that the odor evaluation was significantly different between the control group (2.73 ± 0.62) and the ASD-High DT group (2.04 ± 0.94) [coefficient estimate: 2.34, standard error (SE): 0.89, *p* = 0.009], and a tendency of significant difference between the control group and the ASD-Low DT group (2.13 ± 0.74) (coefficient estimate: 1.85, SE: 1.05, *p* = 0.08).

The odor identification test after the fNIRS measurement session indicated that almost all participants recognized the odor, except three participants in the ASD-High DT group.

### NIRS Results

**FIGURE 5:**
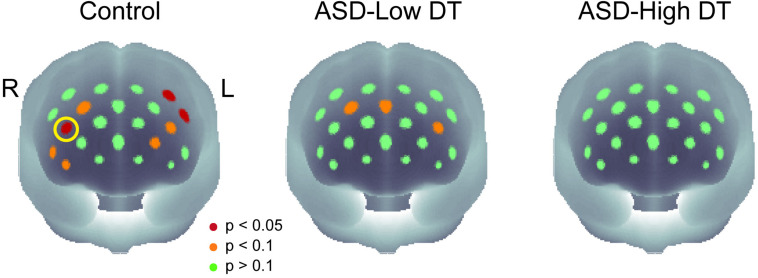
The *p*-maps of the averaged concentration changes in oxy-Hb for the three groups. CH13 that showed significant difference in response between the control group and the ASD-High DT group was marked using a yellow circle. The corresponding brain region of CH13 was estimated as right dorsolateral prefrontal cortex (DLPFC) using virtual spatial registration (Tsuzuki et al., 2007).

**FIGURE 6:**
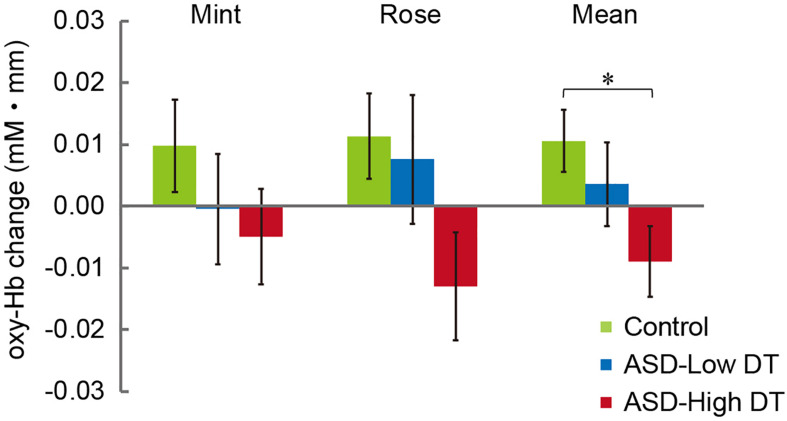
Averaged oxy-Hb responses in CH13 to mint and rose stimuli for the three groups. The results of two-way repeated measures ANOVA showed a significant main effect of group (Control > ASD-High DT). *p < 0.05.

**FIGURE 7:**
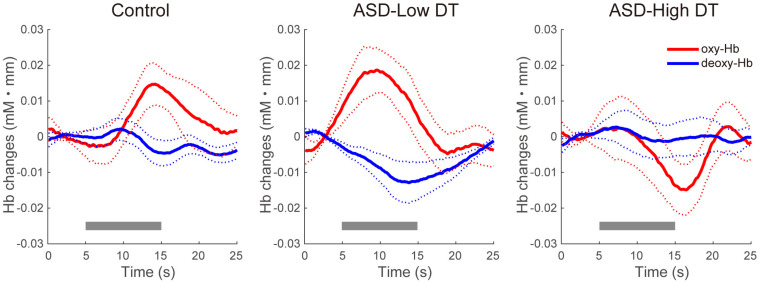
Time series of the averaged concentration changes in oxy-Hb and deoxy-Hb (mM mm) in CH13 for the three groups. The gray bars represent the target period (5 ∼ 15 s). The thick red and blue lines represent the mean concentration changes of oxy-Hb and deoxy-Hb, respectively. The thin dotted red and blue lines represent 1 standard error of concentration changes in oxy-Hb and deoxy-Hb, respectively.

The concentration changes in oxy-Hb and deoxy-Hb were analyzed for each group. Since two-way repeated measures ANOVA with group and odor as factors revealed almost no significant odor effect except for CH6 [*F*(1,35) = 4.32, *p* < 0.05], and no interaction for any channel, the trials in the mint and rose conditions were combined to identify the activated channels in each group (Figure 5) and to create the time course of Hb changes in all channels for each group (Supplementary Figures 2–4). More channels were activated in the control group than in the ASD-Low DT group while there were no activated channels in the ASD-High DT group (Figure 5). Specifically, according to the virtual registration method (Tsuzuki et al., 2007), multiple channels chiefly covering the DLPFC were activated in the control group (CH9, right DLPFC 100%; CH10, left DLPFC 91.7%, FP 8.3%; CH13, right DLPFC 78.9%, FP 21.1%; CH14, left DLPFC 100%; CH17, right DLPFC 50.5%, left DLPFC 39.8%; CH19, left DLPFC 95.1%, FP 4.9%). Similar channels covering the DLPFC were also activated in the ASD-Low DT group (CH10 and CH17). However, no active channel was found for the ASD-High DT group. Among these DLPFC channels, CH9 [*F*(2,35) = 3.49, *p* = 0.042] and CH13 [*F*(2,35) = 3.39, *p* = 0.047] showed a significant main effect of group according to the results of two-way repeated measures ANOVA with group and odor as factors. Particularly, activity of CH13 (right DLPFC) was significantly weaker in the ASD-High DT group than in the control group (*p* = 0.046, Bonferroni correction; Figure 6). The time series of Hb changes in CH13 (right DLPFC) with significant main effect of group (control > ASD-High DT) are shown in Figure 7. The pattern of brain activity of the ASD-High DT group was different from that of the other two groups, and the level of brain activity of this group was weaker than that of the control group. One-way ANOVA showed that there was no significant main effect of group for latency of the oxy-Hb peaks [*F*(2,35) = 0.47, *p* = 0.63], mean amplitude of the deoxy-Hb [*F*(2,34) = 2.41, *p* = 0.11], and latency of the deoxy-Hb peaks [*F*(2, 34) = 1.45, *p* = 0.25]. To exclude the possible influence of perception of odor pleasantness on the observed discrepancy in neural activity between the control and the ASD groups, the rating score of odor pleasantness was included as a covariate of no interest. The results were largely consistent with the analysis without this covariate. Specifically, significant main effect of group was found in CH13 [*F*(2,69) = 3.953, *p* = 0.024], and *post hoc* comparisons with Bonferroni’s correction revealed a significant difference between the control group and the ASD-High DT group (*p* = 0.020). Hence, the group difference in odor-elicited neural activity does not likely come from the difference in perception of odor pleasantness.

**FIGURE 8:**
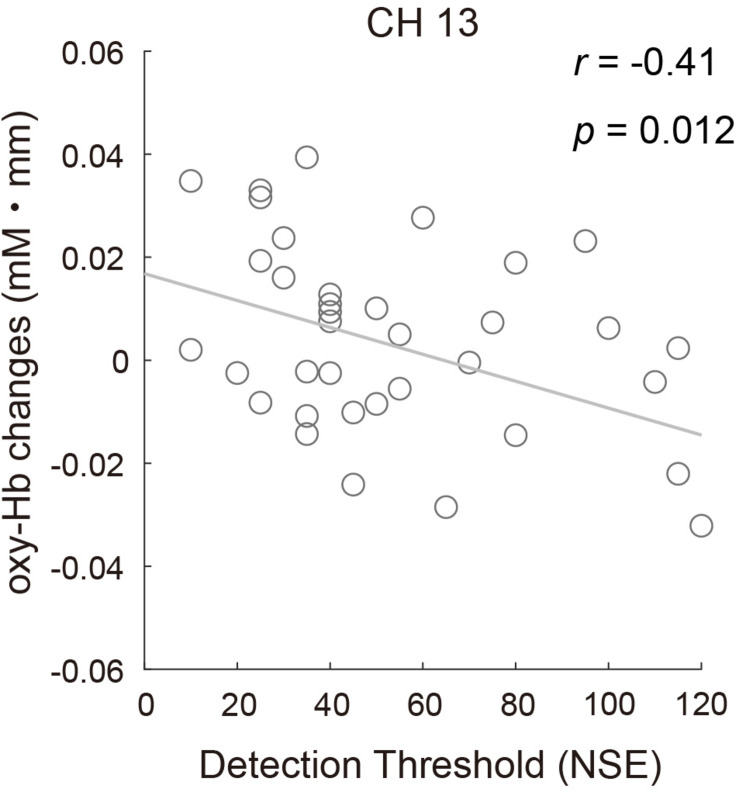
Correlation between the mean concentration changes in oxy-Hb in CH13 and odor detection thresholds for all participants (*r* = –0.41, *p* = 0.012).

To examine whether there was a relationship between odor detection sensitivity and the fNIRS data, the correlation between the oxy-Hb response to the odor in CH13 and the DT of all participants was assessed using Pearson correlation. The result revealed a significant negative correlation (*r* = −0.41, *p* = 0.012; Figure 8), indicating that participants with lower odor detection sensitivity might show reduced brain activity to a certain level of odor stimulation. The relationship between brain activity and other olfactory perception abilities (odor identification and odor evaluation), as well as other sensory characteristics observed from the sensory profile battery, was also evaluated, but no significant correlations were found.

## Discussion

The present study aimed to investigate the neural underpinnings of olfactory processing in individuals with ASD by combining a precise and ASD-friendly olfactory measurement system, which consists of a pulse ejection system, with fNIRS measurement. Significant differences were found between ASD and control groups not only at the behavioral level but also at the neural level. The ASD group with hyposensitivity (ASD-High DT group) to olfactory stimuli was found to be significantly different from the control group with respect to the olfactory DT and pleasant feelings to the used odors. The brain activity in the right DLPFC of the ASD-High DT group was significantly weaker than that of the control group. In addition, the strength of brain activity in the right DLPFC was significantly correlated with the DT in all participants.

### Combined Use of a Sophisticated System for Olfactory Examination and fNIRS

The present study successfully utilized a pulse ejection system in combination with fNIRS to examine neural dysfunction related to olfactory processing in individuals with ASD. The system is unique in that it can precisely control the interval of odor stimulation and, therefore, significantly reduce the potential confounding effect lingering scents have on olfactory perception. Together with an ASD-friendly game-like application for odor detection test, the system is feasible for gauging the olfactory ability of not only high functioning individuals with ASD but also individuals with ASD who have intellectual impairment. While this system has been successfully used for olfactory assessment in children with ASD (Kumazaki et al., 2016, 2018c, 2019), the present study, for the first time tried combining it with fNIRS.

Since some parts of the pulse ejection system were made of metal, it could not be used in fMRI scanning; however, it could be used during fNIRS measurement. Our study verified the feasibility of the combination of this olfactory test system and a highly ecologically valid neuroimaging method such as fNIRS to localize the brain functions of olfactory processing in ASD. The system facilitated the examination of not only high functioning individuals with ASD but also of individuals with ASD who have relatively low intellectual abilities, which has not been well established. This olfactory neuroimaging experimental paradigm is friendly for individuals with mental disorders who have olfactory differences, such as individuals with ASD and schizophrenia, and may also be useful for the future diagnosis of these disorders.

### Olfactory Sensitivity in Individuals With ASD

Our findings showed that participants with ASD with high DT were significantly impaired in their sensitivity and hedonic response to olfactory stimuli but not in their odor identification ability. Previous literature has suggested atypical olfactory function in ASD, but the pattern of findings shows much heterogeneity. This is particularly true for investigations of sensory-driven olfactory function, such as odor detection. Thus, while some studies reported either enhanced (Ashwin et al., 2014) or decreased odor sensitivity (Dudova et al., 2011; Kumazaki et al., 2016) in ASD, most studies reported no significant differences between individuals with ASD and controls in DT (Suzuki et al., 2003; Tavassoli and Baron-Cohen, 2012; Galle et al., 2013; Addo et al., 2017). The discrepancy in findings might come from a complex blend of variance in participants’ demography (age and gender), subtype of ASD (AS, HFA, PDD, etc.), sample size, and the study design/test method used. For instance, most of these studies recruited participants with AS and HFA (Suzuki et al., 2003; Galle et al., 2013; Addo et al., 2017), generally having a higher cognitive functioning than the general ASD population. Therefore, individuals with these subtypes of ASD may have comparable odor sensitivity to that of age-matched TD controls. In addition, though it is common for these studies to include more male participants with ASD (as males are more susceptible to ASD; Baron-Cohen, 2002), their ages varied from children, to teenagers (Dudova et al., 2011; Kumazaki et al., 2016), to young adults around twenties (Galle et al., 2013), to adults in their thirties or forties (Suzuki et al., 2003; Tavassoli and Baron-Cohen, 2012; Ashwin et al., 2014; Addo et al., 2017). While prior evidence reveals that females have higher olfactory functioning (primarily olfactory sensitivity and identification) than males in TD population (e.g., Doty et al., 1984a; Lehrner, 1993; Barber, 1997), the above-mentioned olfactory studies in ASD consistently enrolled more male participants and the gender ratio is therefore not likely to be the source of the discrepancy between studies, instead, age is more likely the source. It is well known that olfactory sensitivity substantially declines due to aging in healthy individuals (e.g., Cain et al., 1995; Schiffman, 1997; Larsson et al., 2000). However, while younger individuals with ASD (<30 years) tend to show lower olfactory sensitivity than TD individuals, older individuals with ASD (>35 years) do not (Larsson et al., 2017). In addition, it is suggested that in TD individuals, olfactory sensitivity develops earlier in life while odor identification ability is acquired later, during adolescence (Tonacci et al., 2017), but this timing could be more heterogeneous and/or shifted in individuals with ASD (Dudova et al., 2011). As for young adults with ASD, who are within our scope, Galle et al. (2013) reported no significant differences in DT between them and TD controls; however, they did show large variability in DT. In addition, this study’s sample size was very small (five individuals with ASD and five controls), which may be the cause of the inconsistencies with our findings.

Considering the similar sample size (over 20) and sampled population (ASD also including individuals with AS and HFA, 10–20 years old), our findings of impaired odor detection ability in young adults with ASD are consistent with the observations of Kumazaki et al. (2016). The observed impaired odor sensitivity in these individuals with ASD might be attributed to age. It is possible that young individuals with ASD might be compromised in their olfactory sensitivity, but the impairment may be alleviated with maturation. This is an open question that warrants further research using longitudinal methods. However, the substantial heterogeneity across studies with respect to odor DT in ASD is in accord with the idea that ASD may be associated with both hyposensitivity and hypersensitivity in olfaction. As such, it is necessary to treat individuals with ASD differently when investigating their relevant brain functions. Therefore, in the present study, we divided participants with ASD into two groups−ASD-Low DT and ASD-High DT−according to their DT and compared their brain responses elicited by odor stimuli with those of age-matched TD controls.

With respect to odor identification, our task did not provide written label alternatives (one target and several foils), as do UPSIT (Doty et al., 1984b) and Sniffin’ Sticks (Hummel et al., 1997), and it was somewhat difficult for the participants to give an answer without any reference information. Even the control group had poor performance in this aspect. This might be the reason for non-significant difference between the groups. Although some previous studies have reported decreased odor identification ability in ASD (Suzuki et al., 2003; Bennetto et al., 2007; Galle et al., 2013; Wicker et al., 2016), there is also evidence of no significant difference compared with TD controls (Brewer et al., 2008; Dudova et al., 2011; Luisier et al., 2015; Addo et al., 2017).

With respect to the hedonic responses to odor stimuli (i.e., pleasantness), the ASD-High DT participants were significantly different from TD controls. This finding is consistent with a previous study on the estimation of odor pleasantness in approximately 10-year-old children with ASD (Hrdlicka et al., 2011). One possibility is that the ASD-High DT group could not process the odor stimuli at a metacognitive level, i.e., self-knowledge of one’s cognitive process (Shimamura, 2008), to rate the pleasantness. In other words, the participants in ASD-High DT group cannot properly rate the pleasantness of odor maybe because they were not fully aware of their cognition of an odor so that they were not able to evaluate the odor as good as the TD controls did. In fact, impaired metacognitive function has been reported in individuals with ASD during perceptual processing of several domains (e.g., Grainger et al., 2016; McMahon et al., 2016). Considering their impaired social capabilities including emotion (Legisa et al., 2013), another possibility could be that participants with ASD had difficulty in emotionally rating an odor.

### Weaker Prefrontal Activity in Individuals With ASD

With respect to the prefrontal fNIRS data, we found significantly weaker hemodynamic responses in the right DLPFC of participants with ASD with lower olfactory sensitivity (the ASD-High DT group) compared to the TD participants. Although there was no significant difference in neural activity between the participants with ASD with relatively higher olfactory sensitivity (the ASD-Low DT group) and the TD participants, the ASD-Low DT group did not show significant right DLPFC activity compared to the control group. These results indicate that individuals with ASD generally have different neuronal basis for processing odor stimuli compared to TD individuals. Such discrepancies may be related to impaired olfactory processing in ASD. More specifically, considering the important role of the DLPFC in executive functions including attention (Duncan and Owen, 2000) and working memory (Levy and Goldman-Rakic, 2000), reduced activity in this region may reflect the general abnormality in attention and working memory in individuals with ASD (Russell, 1997; Travers et al., 2011) during olfactory processing.

In the fNIRS measurement session, although the task requirement was passive perception (i.e., olfaction), the participants were instructed that there could or could not be an odor stimulus during the fNIRS measurement and that they would be asked to report whether they had smelt something after the fNIRS measurement. Therefore, the participants might have attentively monitored their sense of smell, which demanded some degree of attention and working memory. In addition, right DLPFC activation was found to be significantly related to odor sensitivity, which was assessed in the odor DT test that required attention and working memory as well. Thus, it is possible that odor sensitivity indirectly reflects the participants’ attention and working memory, and consequently correlates with the level of brain activity in the region responsible for these functions. From this perspective, the weaker response in the DLPFC of participants with ASD with lower sensitivity (the ASD-High DT group) may be attributed to their deficits in attention and working memory. The neural correlates of olfactory attention and olfactory working memory have been suggested to be in the olfactory bulb or the piriform cortex (Keller, 2011) and in the primary olfactory cortex (Zelano et al., 2009), respectively. On the other hand, robust involvement of the DLPFC in olfactory working memory has been demonstrated in one positron emission tomography (PET) study (Dade et al., 2001), while another fNIRS study also reported the engagement of the bilateral DLPFC and FP in response to an olfactory task requiring attention and working memory (Takakura et al., 2011). Similarly, the significant channel (CH13, virtually covering DLPFC 78.9% and FP 21.1%) of our study also partially covered the FP, which is suggested to function as “gateway” that biases attention (Burgess et al., 2005; Gilbert et al., 2006; Takakura et al., 2011).

The question remains as to how altered working memory and attention in ASD are associated with the olfactory anomaly. There are two possible explanations, one is at the perceptual level and the other is at the cognitive level. The perceptual explanation is that individuals with ASD may have impairments in sensory processors such as in the olfactory bulb and/or olfactory cortex, which produce weakened perceptual signals, which may then be reflected as impaired olfactory working memory. The cognitive explanation posits that perceptual processing of individuals with ASD may not be problematic; instead, perceptual signals may not be processed efficiently to be held as explicit meta-cognitive signals in the DLPFC. In other words, even if individuals with ASD may have unconsciously processed the odors to a similar sensory degree as TD individuals, they may not consciously process the odors due to the problems of attention and/or working memory. This imprecise operation may lead to compromised olfactory processing in ASD.

The cognitive explanation may, however, be the most likely. One reason is that the DLPFC may not activate as a function of the intensity of olfactory stimulation, instead, it may primarily encode the presence or the type of olfactory stimuli at the conscious (i.e., cognitive) level. During the fNIRS measurement, we used an odor stimulus with an intensity of 120 NSE, which is higher than the DT of almost all participants and, therefore, should have been perceived. This is also validated by the participants’ performance in the post-experiment odor identification task: none of the 12 participants in the ASD-Low DT group failed to recognize the odor, and only 3 of the 13 participants in the ASD-High DT group failed to explicitly recognize the odor. Thus, the significant difference in neural responses between the ASD-High DT group and the control group is not likely caused by low-level sensory processing of odor stimuli but might possibly be due to higher-order olfactory processing, such as executive function, which has been shown to be impaired in ASD (Hill, 2004; Kenworthy et al., 2008). Moreover, while there was no statistically significant difference in brain activity between the ASD-Low DT group and the control group, the brain activity of the ASD-Low DT group showed a similar but weaker pattern compared to the control group (Figure 5). Considering that the two groups have almost equivalent olfactory sensitivity, the odor used should have stimulated their olfactory system to a comparable degree. However, the ASD-Low DT group showed weaker neural responses. This group may have a problem in odor processing in more complicated social situations that require a higher load of olfactory working memory and/or attention.

A precise definition of the DLPFC’s contribution to olfactory working memory and attention is not established. On the one hand, participants with ASD might not be able to activate the DLPFC in response to olfactory stimuli as efficiently as the control group. On the other hand, they may recruit brain regions other than the DLPFC in situations requiring olfactory working memory and/or attention. A future systematic examination of the relationship between working memory and attention and DLPFC activity in individuals with ASD is necessary to investigate these possibilities. In any case, DLPFC deficit as a cause of olfactory processing plausibly explains age dependent olfactory sensitivity for individuals with ASD: Prefrontal cortex develops rapidly during adolescence and that facilitates matured DLPFC function, resulting in unimpaired olfactory function in adults with ASD.

There are also some other DLPFC-related issues worth considering. For instance, a recent genetic study reported decreased olfactory receptor expression in the DLPFC of individuals with chronic schizophrenia (Ansoleaga et al., 2015). The authors suggested that the deregulation of olfactory receptors is associated with olfactory alterations in these patients. Whether there is a similar mechanism in individuals with ASD remains unclear and would be interesting to investigate in future studies. Additionally, while the present study found a significant group difference in the DLPFC of the right hemisphere only, it does not necessarily indicate that the right DLPFC is predominantly involved in olfactory working memory; however, it may contribute to the ongoing research on the lateralization of the working memory of various modalities in the DLPFC (Smith et al., 1996; Murphy et al., 1998) and the lateralization of olfaction-related processing (Zatorre et al., 1992; Brand et al., 2001; Ishimaru et al., 2004b). Nevertheless, the interpretation of reduced DLPFC activity as a result of impaired odor sensitivity should be treated with caution, since the olfactory task in the odor DT test session and fNIRS recording session differed due to their respective purpose.

Finally, differences in IQ among groups in relation to fNIRS results and DT should be discussed. As for the fNIRS results, one may ask whether the weaker prefrontal activity of the participants with ASD is due to their impaired ability, as reflected by IQ, to properly engage in the fNIRS experiment. Although the participants with ASD had lower IQ than the TD control group (Table 1), they had no problem in participating in the experiment at any stage as confirmed by their proper verbal responses to the odor identification and evaluation questions after each fNIRS measurement session. In addition, they did not have any difficulty in performing the odor DT task, which was more complicated than the fNIRS task. Another IQ-related issue is that perceptual sensitivity to odor may directly relate to IQ as our ASD-Low DT group had higher IQ than ASD-high DT group. However, this is not likely because no correlation between IQ and olfactory processing has been reported in the general population (Hedner et al., 2010). On the other hand, since performance on IQ tests depends on executive functions including attention, the lower IQ of the participants with ASD may indirectly reflect their deficits in directing attention toward odor stimuli, which may have led to their impaired olfactory processing and weaker/absence of prefrontal activity.

### Limitations

Although our sample size was comparable to that of previous studies on olfactory function in ASD, considering the high degree of heterogeneity of ASD, it was still not sufficiently large. This may be one of the reasons why we did not have robust statistical results. Another limitation is that the present study included individuals with ASD with several different subtypes (i.e., AS, AD, and PDD-NOS). Therefore, we did not reveal subtype-specific olfactory traits in ASD. However, since most previous studies on olfactory dysfunction in individuals with ASD predominantly included individuals with AS and HFA, who are more capable of performing experimental tasks, the present study contributes to the limited evidence on behavioral and neural impairments in individuals with ASD with moderate to severe intellectual difficulties, who can also undergo both psychophysical and neuroimaging measurements with our ASD-friendly system. A more systematic study of olfactory processing in ASD with a larger sample size and taking into account various subtypes of ASD is warranted. In addition, we used odor of mint, an olfactory-trigeminal stimulus, while we did not assess the intranasal trigeminal sensitivity of the participants. We tried using many odors in the preliminary experiments and odors of rose and mint were found to be the most suitable for obtaining consistent brain responses from participants as measured with fNIRS. The present findings with the usage of odor of mint should not be simply generalized to odor stimuli as a whole. For instance, the mint odor induced relatively smaller difference in brain activation between the ASD-Low DT and the control groups regardless of their comparable DT values, although these are not statistically significant. Future study could examine odor-type-dependent cerebral responses, particularly the difference in responsiveness to odors stimulating the trigeminal nerve or not.

## Conclusion

The present study verified the feasibility of combining a precise and easy-to-use system for olfactory measurement−the odor pulse ejection system−with fNIRS. Using this ASD-friendly system, we successfully measured olfactory function in individuals with ASD, including those with moderate to severe intellectual impairment. Compared to TD controls, participants with ASD with lower odor sensitivity showed blunted activity in the right DLPFC in response to odor stimulation. Even in participants with ASD with normal odor sensitivity, DLPFC activities were not as significant as in the control group. In addition, the strength of olfaction-evoked neural activity in the right DLPFC was correlated with DT. These findings indicate that differential DLPFC function for olfactory processing, particularly olfactory working memory and/or attention, is related to odor-processing anomaly in ASD. The present study provides insight into the neural mechanisms of specific olfactory malfunctions associated with ASD by revealing deficit in the cognitive brain function as a possible cause. Future establishment of fNIRS-based biomarkers might facilitate the use of a non-invasive technique in identifying olfactory difficulties in individuals with ASD.

## Data Availability Statement

The dataset generated for this study are available, under the restriction of ethic permission obtained from our university, on request to the corresponding author.

## Ethics Statement

The studies involving human participants were reviewed and approved by the ethics committee of the Keio University, Faculty of Letters (No. 16028). The patients/participants provided their written informed consent to participate in this study.

## Author Contributions

NN and MX analyzed the data. NN, MX, and YM interpreted the data. MX and YM wrote the manuscript. NN, YM, HK, and KO designed the study. NN and YM performed the experiment. All authors contributed to the article and approved the submitted version.

## Conflict of Interest

The authors declare that the research was conducted in the absence of any commercial or financial relationships that could be construed as a potential conflict of interest.
