# Supplementary material for: Prefrontal Responses to Odors in Individuals With Autism Spectrum Disorders: Functional NIRS Measurement Combined With a Fragrance Pulse Ejection System
Source: Front Hum Neurosci. 2020 Oct 8;14:523456. doi: 10.3389/fnhum.2020.523456 (PMC7579723; doi:10.3389/fnhum.2020.523456)
Supplement: Supplementary file 1 [file Table_1.DOCX]

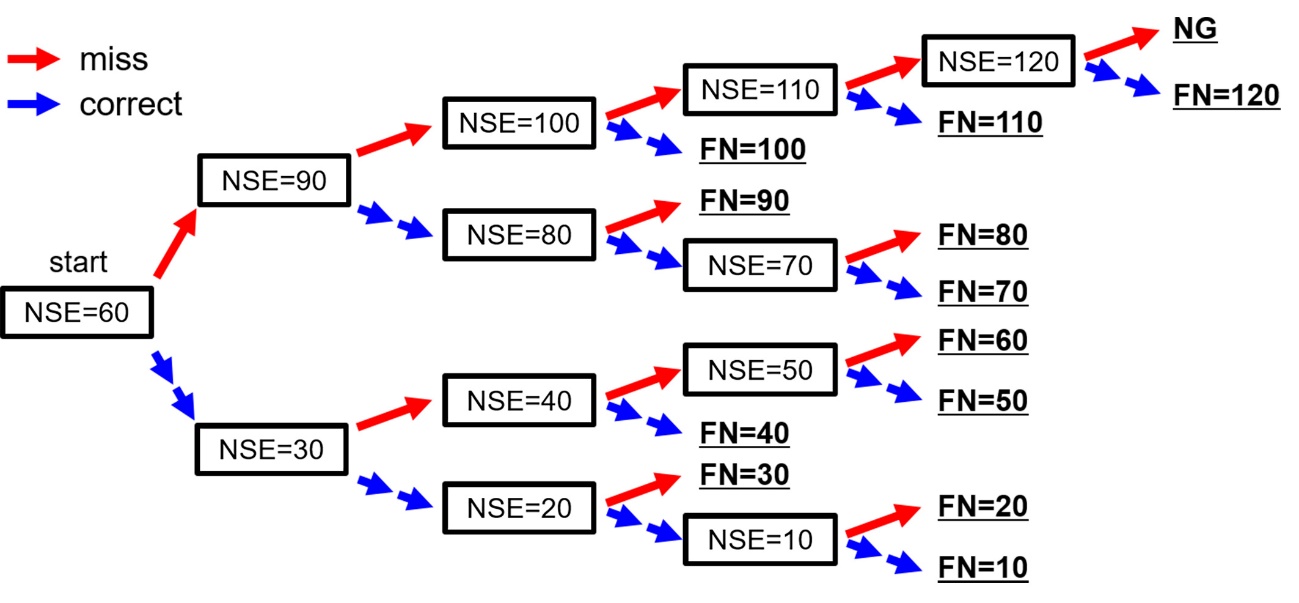


Supplementary Figure 1. Schematic diagram of the algorithm used for the odor detection threshold (DT) measurement. First, an NSE of 60 was used. The NSE was increased if the answer was wrong, and the NSE was decreased if the answer was correct for two consecutive trials. For the first trial only, when a participant made an error (selecting a box that contained an odorless stimulus), the NSE increased by 50%; once two consecutive trials were successful, the NSE decreased by 50%. For the rest of the trials, the increment or decrement unit of the NSE was 10. The maximum and minimum values of NSE were 120 and 10, respectively. The detection threshold was generated after the procedure was completed. NSE: the number of simultaneous ejections, FN: final number.


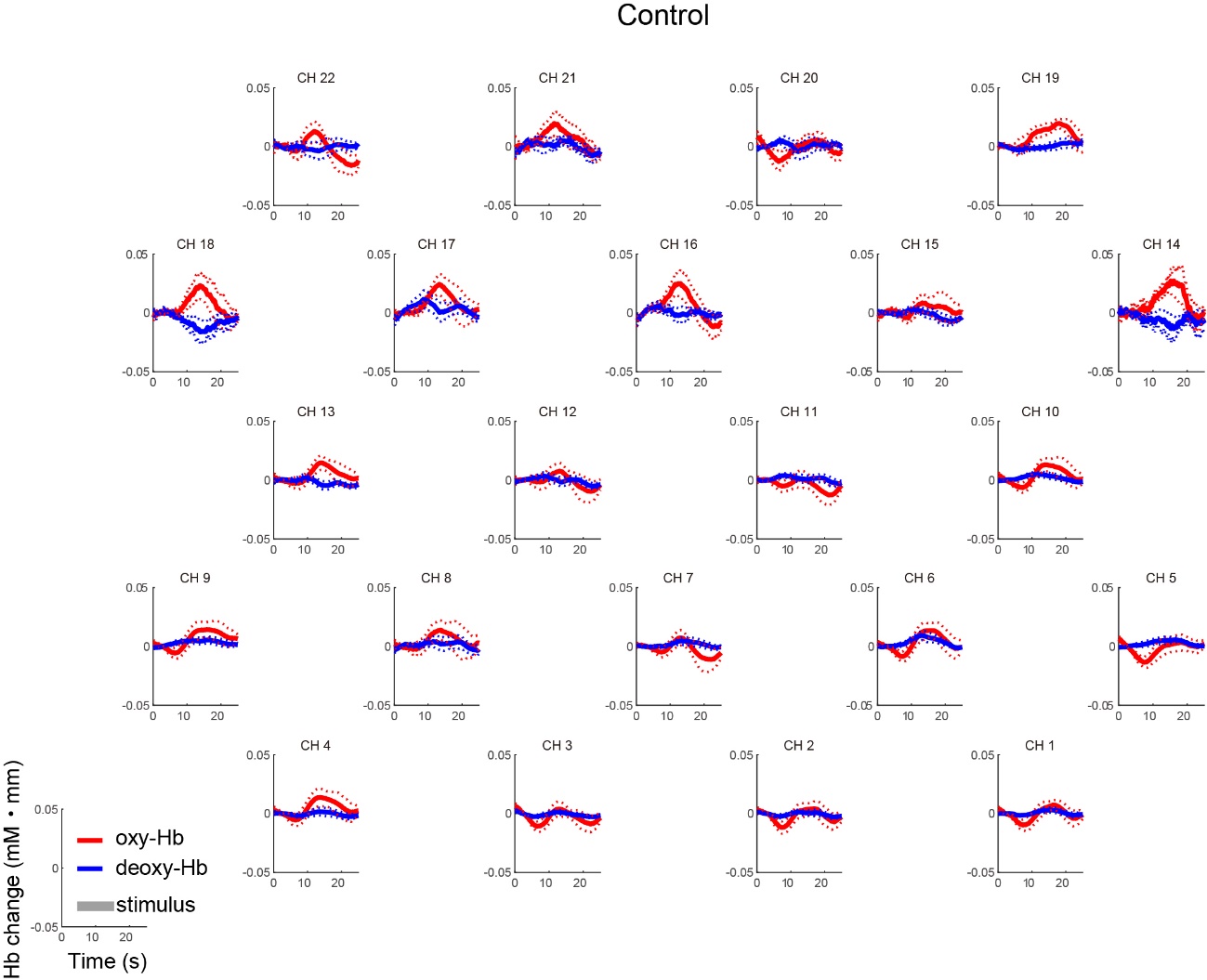


Supplementary Figure 2. The time series of oxy-Hb and deoxy-Hb in all channels for the control group. The dotted lines represent 1 standard error.


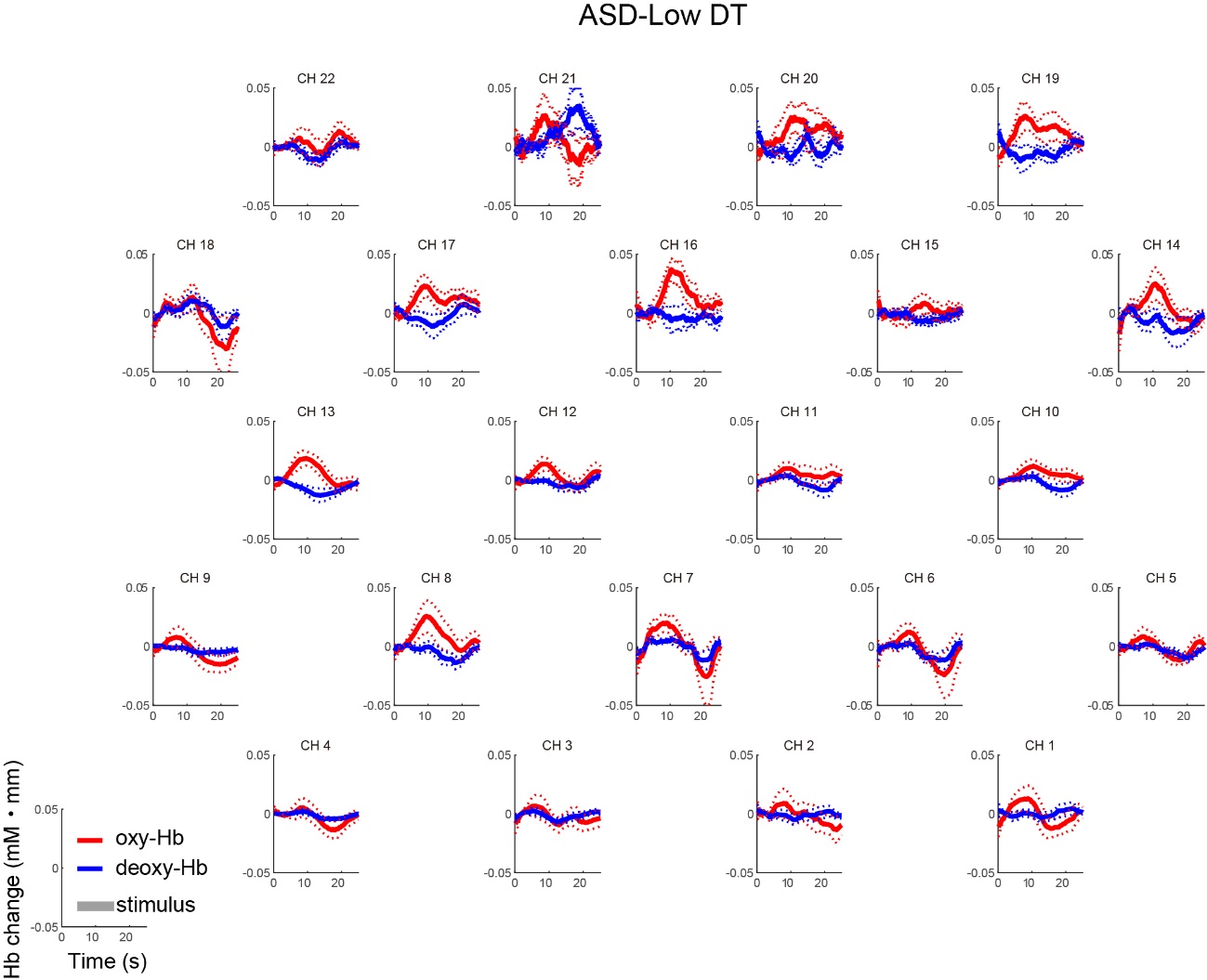


Supplementary Figure 3. The time series of oxy-Hb and deoxy-Hb in all channels for the ASD-Low DT group. The dotted lines represent 1 standard error.


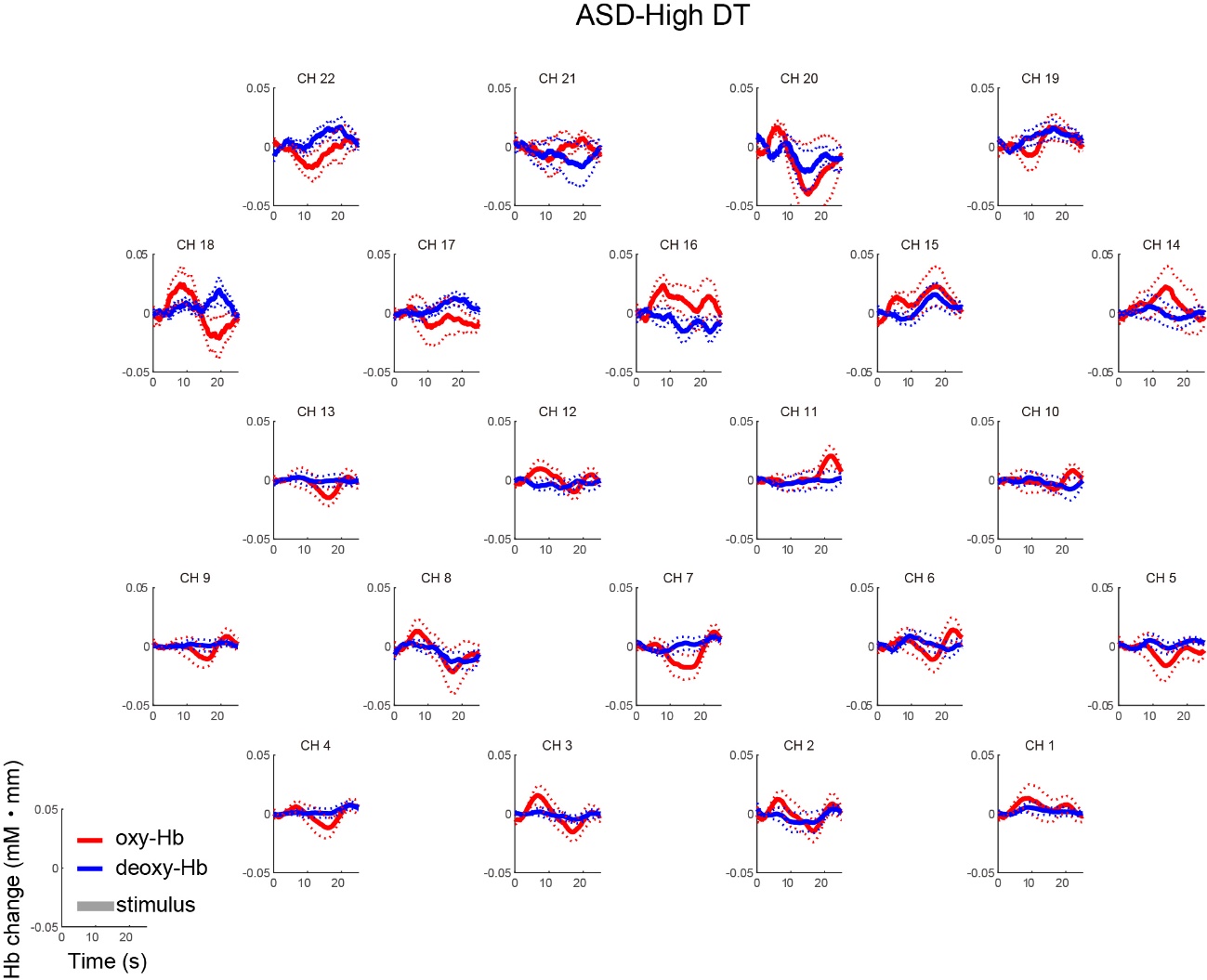


Supplementary Figure 4. The time series of oxy-Hb and deoxy-Hb in all channels for the ASD-High DT group. The dotted lines represent 1 standard error.
